# A Surface Imprinted Polymer EIS Sensor for Detecting Alpha-Synuclein, a Parkinson’s Disease Biomarker

**DOI:** 10.3390/mi15020273

**Published:** 2024-02-15

**Authors:** Roslyn Simone Massey, Rishabh Ramesh Appadurai, Ravi Prakash

**Affiliations:** Department of Electronics Engineering, Carleton University, 1125 Colonel By Drive, Ottawa, ON K1S 5B6, Canada; roslynmassey@cmail.carleton.ca (R.S.M.); rishabhappadurai@cmail.carleton.ca (R.R.A.)

**Keywords:** surface imprinted polymers, electroimpedance spectroscopy, label-free biosensors, Parkinson’s disease, α-synuclein

## Abstract

Parkinson’s Disease (PD) is a debilitating neurodegenerative disease, causing loss of motor function and, in some instances, cognitive decline and dementia in those affected. The quality of life can be improved, and disease progression delayed through early interventions. However, current methods of confirming a PD diagnosis are extremely invasive. This prevents their use as a screening tool for the early onset stages of PD. We propose a surface imprinted polymer (SIP) electroimpedance spectroscopy (EIS) biosensor for detecting α-Synuclein (αSyn) and its aggregates, a biomarker that appears in saliva and blood during the early stages of PD as the blood-brain barrier degrades. The surface imprinted polymer stamp is fabricated by low-temperature melt stamping polycaprolactone (PCL) on interdigitated EIS electrodes. The result is a low-cost, small-footprint biosensor that is highly suitable for non-invasive monitoring of the disease biomarker. The sensors were tested with αSyn dilutions in deionized water and in constant ionic concentration matrix solutions with decreasing concentrations of αSyn to remove the background effects of concentration. The device response confirmed the specificity of these devices to the target protein of monomeric αSyn. The sensor limit of detection was measured to be 5 pg/L, and its linear detection range was 5 pg/L–5 µg/L. This covers the physiological range of αSyn in saliva and makes this a highly promising method of quantifying αSyn monomers for PD patients in the future. The SIP surface was regenerated, and the sensor was reused to demonstrate its capability for repeat sensing as a potential continuous monitoring tool for the disease biomarker.

## 1. Introduction

Neurological disorders are the leading cause of disability worldwide, affecting 15% of people, with neurodegenerative diseases such as Parkinson’s disease (PD) and Alzheimer’s disease currently accounting for 31–36% of neurological disorders [1]. The prevalence of neurodegenerative disease is rising [2], yet despite the rapidly aging population, there is limited access to neurological healthcare and accessible diagnostic tests [3]. At present, neurodegenerative diseases are mainly diagnosed by neurological and physical exams [4]. However, observable symptoms occur years or even decades after the onset of disease pathology. In order to detect neurodegenerative diseases in their earliest state, early identification of pathological biomarkers could potentially be a powerful tool.

α-Synuclein (αSyn) is a neural protein with remarkable conformational plasticity in its physiological form, fulfilling multiple roles in the body [5,6]. However, when misfolded and/or phosphorylated, αSyn becomes pathological and aggregates into fibrils, leading to synucleinopathies such as PD [7]. Aggregation of αSyn and subsequent neurodegeneration of midbrain dopaminergic neurons produces the loss of motor symptoms used for the initial diagnosis of PD [8]. Pathological αSyn aggregation and fibril formation occur years before the expression of clinically significant symptoms. Once PD is suspected, the diagnosis can be confirmed with cerebral spinal fluid (CSF) seeding activity testing, which measures the rate at which αSyn forms toxic aggregates [9]. Unfortunately, this test cannot be used as a screening tool for early detection of PD as it is highly invasive, requires specialized laboratory equipment, and can take from 5 to 13 days to perform these tests [10]. There are a few promising examples of biosensing platforms suitable for less invasive, less cumbersome, and hence more accessible αSyn quantification, such as our organic electrolyte-gated FET aptasensor platform and Adam et al.’s electrochemical biosensor [11,12] to list a few. These emerging biosensors rely on a bioreceptor molecule, either an aptamer or an antibody, adding unique complexities to sensor shelf-life and usability as a continuous monitoring device.

As an alternative to electrochemical and electrolyte-gated biosensors, electroimpedance spectroscopy (EIS) sensors transduce sample target biomarker binding by measuring the change of reactance and resistance as a function of angular frequency [13]. EIS is capable of rapid, non-destructive, label-free characterization without current production to perform measurements [14]. EIS is highly sensitive to near-surface effects, making it ideal for affinity biosensors, with a simple electrode design requiring only interdigitated structures of counter and reference electrodes. Conventional EIS biosensors rely on changes resulting from enzymatic reactions facilitated by gold nanoparticles or selective binding action in the presence of a bioreceptor such as an antibody or aptamer (short oligomer DNA chains). Di Mari et al. have produced a zinc oxide nanowire-based EIS sensor that is made sensitive to αSyn using antibodies and amplified with gold nanoparticles [15]. Their devices showed a promising linear range of 0.5–10 pg/mL in plasma. Challenges with their approach are the sensitivity of electrochemical EIS sensors to surface effects [16].

EIS, combined with affinity-based recognition, is a facile, rapid, and exceptionally durable platform for biosensing [16,17]. Synthetic affinity recognition methods focus on cost-effective fabrication processes for highly selective and repeatable target binding. Conventional immunoassays, the gold standard of biomolecule quantification, rely on the selective binding of immunoglobulins (antibodies; Ig) [17]. These biologically sourced materials are highly sensitive to the environment and fabrication processes, which makes integrating them into commercial biosensors challenging. In contrast, synthetic ‘antibody mimics’ like surface imprinted polymers (SIPs) possess excellent thermal and chemical resistance despite low-cost fabrication processes.

SIPs are polymers imprinted with a target biomolecule to form three-dimensional stereo cavities that bind the target with high specificity. Molecular imprinting and stamp imprinting are the most commonly reported methods of fabricating SIP [18]. In molecular imprinting, a monomer is polymerized, or a polymer is crosslinked around a biomarker target. Yang et al. produced a P-glycoprotein SIP with a LoD of 22 fg/L. However, this approach faces the challenges of on-surface cross-linking [19]. Polymerizing and crosslinking reagents can interfere with the biomarker structure, whilst milder processes, such as UV cross-linkable materials, are often water soluble [14]. Stamp imprinting avoids this by creating a nanoimprint on an already deposited layer through the stamping process [18]. Werner et al. compared on-surface polymerization to stamp imprinting for SIP using *Escherichia coli* cell as a template [20]. Atomic Force Microscopy (AFM) imaging of the imprinted polymers showed smooth surfaces and the presence of stereo cavities for detection [20]. Pressing biomarker targets into a polymer has shown to be an effective method for producing SIP affinity surfaces for targets as large as cells down to nanoscale targets as small as ions [21].

In this work, we report a highly specific EIS biosensor combined with a SIP nanomaterial as a bioreceptor for simple and rapid quantification of αSyn. The SIP was prepared using a stamp imprinted with polycaprolactone (PCL). PCL is a solution-processable, biocompatible, biodegradable polymer with a dielectric constant of 3.2 [22,23]. PCL melting point is 60 °C, low enough to minimally affect lyophilized proteins. PCL does not dissolve in water or swell (less than 0.25% swelling over 10 h [24]), making it robust. In our previous work demonstrating a proof-of-concept PCL SIP EIS biosensor, we implemented a thermally pressed PCL SIP layer over interdigitated electrodes (IDEs) on a passivated silicon substrate. The stamp used for imprinting consisted of αSyn on polydimethylsiloxane (PDMS) [25]. We were able to demonstrate concentration-dependent EIS behavior but with significant challenges. The first was the fabrication process, which relied on thermal pressing, leading to a thick, non-uniform PCL SIP layer ranging between 10 µm and 200 µm. The high thicknesses contributed to the low resolution between concentrations. The PDMS αSyn stamp had highly variable material density due to the hydrophobic nature of the polymer, and the adhesion to PCL led to damage and low device success rate. Poor biomarker distribution and density on the stamp can impact the signal-to-noise ratio of the devices and produce significant device-to-device variation. We have greatly improved the device structure and fabrication process to produce a much more sensitive and robust biosensor. We used a solution-processed PCL to control the layer thickness and a novel PVA stamp to improve the biomarker distribution. The resultant biosensor can detect low levels of αSyn in tested solutions. In order to minimize the concentration-dependent signal, we tested the sensitivity of the device with solutions of 1 µg/mL, with a varying ratio of αSyn and a homologous control biomolecule ß-synuclein (ßSyn). ßSyn was selected as a control material as it is a synuclein neural protein that has a similar primary structure to αSyn with slight structural differences (αSyn has 140 amino acids whereas ßSyn has 137) [26]. αSyn is more prone to agglomeration due to its different charge distribution and shape. Testing of αSyn and ßSyn combinations showed the PCL SIP device has a linear range of 5 µg/L to 5 pg/L. With an integrated PCL microfluidic channel, the linear range demonstrates the same concentration-dependent behavior. Overall, we have produced a novel SIP EIS biosensor with a facile, scalable fabrication process leveraging low-temperature processing.

## 2. Materials and Methods

### 2.1. Device Fabrication

**Figure 1:**
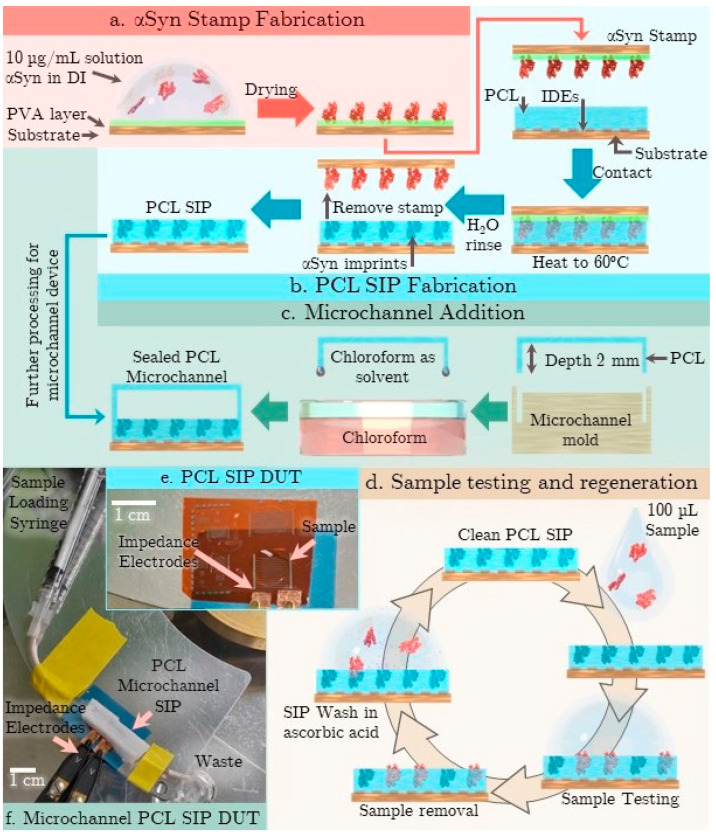
(**a**) aSyn Stamp fabrication process (**b**) SIP fabrication on IDEs (**c**) microfluidic channel addition method (**d**)Sample testing and regeneration process (**e**) Photograph showing PCL SIP device under test (DUT) (**e**) EIS IDE prior to PCL deposition (**f**) A microfluidic channel integrated SIP EIS DUT.

Stamp: 400 nm of polyvinyl alcohol (PVA) is a static deposition spin-coated from a 10 wt.% solution of PVA in chloroform onto a glass slide. The stamp was formed by dropcasting 50 µL of 1 mg/mL αSyn in deionized water (DI) onto a 0.5 cm^2^ area of the PVA and dried at room temperature for 2 h (Figure 1a).

PCL SIP: Kapton substrates (500 EN, Dupont, Wilmington, DE, USA) are patterned with 100 nm of Aluminum (Al) and 100 nm of chromium (Cr) using standard lift-off lithography techniques. The interdigitated electrodes (IDE) area was 25 mm^2^, formed by 20 fingers with a measured gap of 102 µm and width of 153 µm (Figure 1b). A 600 nm thick layer of PCL is deposited on the IDEs by dynamic deposition spin coating (at 6000 rpm). The αSyn stamp was placed in contact with the PCL surface, weighted with a 200 g mass, and heated to 60 °C for 2 min. Once cool, the structure was submerged in water, allowing the PVA stamp polymer to dissolve and releasing the EIS device without damage. Any remaining template biomaterial was removed by successive washes with 0.5 mM ascorbic acid and deionized water (DI) to ensure complete αSyn removal.

PCL microfluidic channel: A microfluidic channel was made by melting PCL into a mold (Figure 1c). The well depth was 2 mm deep, and luer lock tubing was melted into the microfluidic channel for simple sample loading and waste removal. The microfluidic channel was then adhered to the PCL microfluidic channel using chloroform as a solvent. The PCL of the microfluidic channel and the surface form an excellent seal after the chloroform of gases, leaving an integrated sample handling method.

### 2.2. Sample Preparation

Test samples were created by 10-fold serial dilutions from 10 mg/L to 100 ag/L of αSyn in DI. Constant ionic concentration solutions were created using αSyn and ßSyn in varying ratios to produce solutions of serially decreasing αSyn solutions but with a constant total ionic concentration. The concentrations of αSyn in these solutions were 10-fold dilutions from 50 µg/L down to 100 ag/L.

αSyn monomer and ßSyn were supplied by the Laboratory for Aptamer Discovery and Development of Emerging Research (LADDER) group in the Chemistry Department, Carleton University. Test materials were stored at −20 °C and vortexed before testing to avoid undesirable material aggregation.

### 2.3. Testing Processes

The Agilent 4294A impedance analyzer (Agilent Technologies, Inc., Santa Clara, CA, USA was used for data collection. The impedance magnitude and phase angle were collected over a logarithmic frequency sweep from 40 Hz to 100 MHz with an amplitude of 500 mV. For each solution, 10 µL of sample is incubated on the surface for 1 min prior to testing. Each data collection was repeated three times. The surface is then rinsed with DI, followed by 0.5 mM ascorbic acid, and a final DI rinse and N_2_ drying to ensure all material is removed from the surface between tests. The device is then ready for the next test. TAE (1×) buffer is tested throughout each testing session as a baseline for evaluating the sensor drift over time, normalizing between devices, and visualizing the efficacy of the device regeneration.

Parameters were extracted from Nyquist plots where the collected impedance magnitudes and phase angles were used to calculate the real (Z′) and imaginary (Z″) components for further analysis. Experimental data was analyzed using MATLAB.

### 2.4. Surface Profile Characterization of the PCL SIP Layer

**Figure 2:**
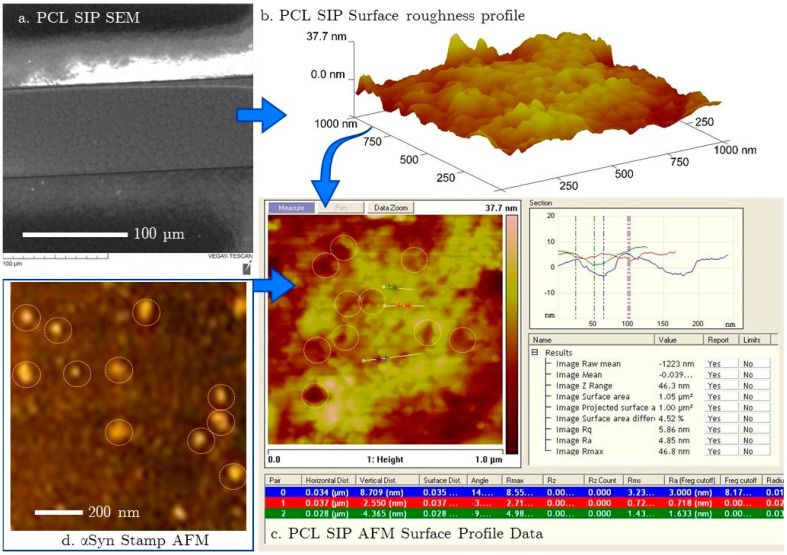
(**a**) SEM image of the PCL SIP on IDE electrode (**b**) AFM image of PCL SIP after testing and regeneration (**c**) AFM micrograph showing surface profile characteristics of the SIP in sensing area (**d**) AFM micrograph of the αSyn stamp showing variable sizes of immobilized material.

Scanning Electron Microscopy (SEM) was performed with a Tescan Vega-II XMU VPSEM (TESCAN, Kohoutovice, Czech Republic). Figure 2a shows the SEM topography of a 238.1 µm by 238.1 µm scan of a SIP on an EIS electrode post-testing and regeneration.

The scale-like appearance of the PCL is a factor of the heat-melt process involved in the stamping process. The important observation here is the size of the crystals formed. Without the presence of a stamp, we observe crystals on the scale of 100 µm–mm. In the presence of the stamp, we observe significantly smaller crystals (scale of 2–5 µm) formed by the stamp protein acting as nucleation points.

A diameter of 2.8 nm is estimated for αSyn monomers (based on the partial specific volume of a globular protein, where the approximate volume is calculated as 1.2 times the protein mass in Daltons [27]). We expect the surface cavities to be in this range for single αSyn monomers, which we further examined using atomic force microscopy (AFM).

AFM images were collected using a Veeco Dimension 3100 AFM (Veeco, Plainview, NY, USA) in tapping mode. Nanoscale AFM lateral resolution is dependent on tip sharpness and profile, causing lateral feature size to be inflated by rough surfaces or adjacent particles. z dimension deflection is a reliable indicator of feature size. Figure 2b shows a 1 µm by 1 µm AFM scan of a PCL SIP after testing and surface regeneration. The average surface roughness, *Rq*, for the surface image in Figure 2c was 5.86 nm, with an image root mean square error, *Ra*, of 4.85 nm. Measured cavities range from the smallest values of less than 2 nm to larger cavities almost 15 nm deep, indicative of αSyn agglomeration on the surface stamp. Figure 2d shows a 1 µm by 1 µm scan of a comparable area of the αSyn stamp on glass, illustrating the comparable material sizes (z dimension of molecules were between 3 and 9.2 nm), and the material size variation confirms that there is some anticipated agglomeration in the αSyn stamp. Thus, the imprints on the PCL SIP shown in Figure 2b,c are consistent with the sizes of the observed dried αSyn of the stamp. The surface cavities demonstrate effective stamp imprinting of the PCL sensing surface.

## 3. Results and Discussion

### 3.1. Impedance Spectroscopy Data Analysis

**Figure 3:**
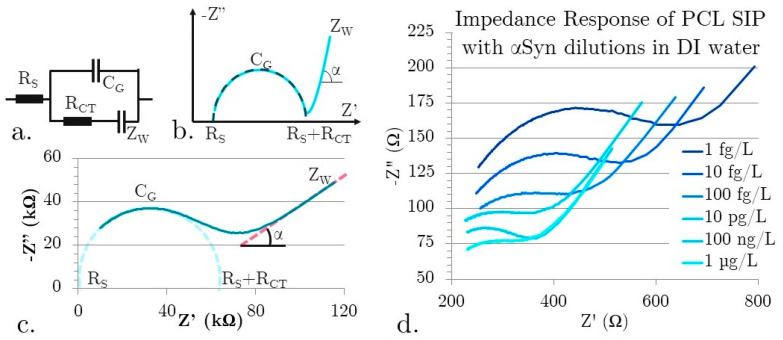
(**a**) Randles-Ershler circuit model (**b**) Equivalent circuit data shape and parameter extraction (**c**) Real sensor data example and extraction locations for Randles-Ershler behavior (**d**) EIS response of real data for dilutions series of αSyn in DI.

Five separate SIP EIS biosensors were fabricated and tested. Each test was repeated three times with each test solution. Given that the EIS biosensor tests electrolytes, it is suitable to analyze the impedance response data with a Randles-Ershler equivalent circuit model (Figure 3a) [28]. Figure 3b shows the expected graph shape from the Randles-Ershler Nyquist plot. This is a basic model that is applied to both faradaic and non-faradaic EIS biosensors. Faradaic biosensors are defined as having a redox species that generates a charge. Non-faradaic biosensors do not rely on charge generation and are generally label-free. It is important to note, though, that there is not necessarily a direct correspondence between circuit elements and underlying physical processes; for example, the simplified Randles-Ershler circuit model lumps the entirety of the sensing mechanism processes into a single element, C_G_.

There are four main parameters: R_S_ or solution resistance, C_G_ or geometric capacitance, Z_W_ the Warburg element, and R_CT_, the charge transfer resistance. The prevalence of the elements is dictated by the device’s architecture and materials. Solution resistance (R_S_) is dependent on the finite ion conductance of the bulk solution [29]. Therefore, it is affected by concentration but not by binding processes. The Warburg impedance, Z_W_, is usually physically insignificant in non-faradaic biosensors, as it is a delay arising from the diffusion of electroactive species to the electrode. Thereby, it only has an appreciable effect at low frequencies and is affected by material transport processes such as convection. The ideal Warburg element has a phase shift of −45°. R_CT_ captures two effects: the energy barrier to redox species (caused by electrostatic repulsion or steric hindrance) and the overpotential. In non-faradaic EIS biosensors, it also models the leakage current from imperfect insulator dielectrics.

C_G_ is the capacitance between the electrodes and the electrolyte solution. It can be modeled as a series of capacitances, including surface insulators, double-layer capacitances, and surface modifications. The electric double layer (EDL) is created by the alignment of charged materials in solution to electrodes of opposite charge. Thus, electric fields in ionic solutions decay exponentially because the alignment of ions negates the effective field. The decay distance from the solid-liquid interface is called the Debye length and is proportional to the square root of ion concentrations. Another contributor to the C_G_ is the adsorbed molecules on the surface. In the absence of charge production, C_G_ is the dominant capacitance term. The C_G_ also contains a constant phase element that dominates at low frequency and can account for the complex double-layer capacitance of the remaining fluid on the surface, adsorbed molecules, and porous surface architectures.

(1) $$Z\left(\omega \right)=\frac{V\left(t\right)}{I\left(t\right)}=\frac{V_{0}\sin\left(\omega t\right)}{I_{0}\sin\left(\omega t+\varphi \right)}\;\;Z^{\prime }=\left|Z\right|cos\varphi \;Z^{\prime \prime }=\left|Z\right|sin\varphi$$

(2) $$C_{G}=\frac{1}{\omega_{peak}R_{CT}}\;\;\;\;\;\;\;\;\;Z_{W}=\frac{1}{\omega |Z|}$$

In the ideal situation of non-faradaic biosensors, R_CT_ would be theoretically infinite as no charge would be crossing the perfect insulator. However, due to the polarizability of polymers and confirmational changes in materials, R_CT_ is finite. Under these conditions, the imaginary portion of the impedance is inversely proportional to the EDL capacitance [30]. This creates an incomplete semicircular shape with a slow transition to linear behavior, even in non-faradaic biosensors. This deviation from the ideal can be attributed to surface non-uniformity, roughness, and porosity. These kinds of surface effects can create sub-microscopic areas, each with a unique resistance-capacitance contribution to the overall behavior. Parasitic impedances and frequency dispersion—the transformation of dielectric response from one mode of polarization to another—are usually described in the *Z*_W_ [31]. As the sensing mechanism derives from changes in near-surface effects and particularly the double layer capacitance in the geometric capacitance, repeatable C_G_ extraction is imperative. The Randles equivalent circuit model and parameter extraction methods have been established in the literature for approximating the non-faradaic biosensors.

The x-intercepts of the semi-circle shown in Figure 3b represent the surface resistance (R_S_) and contact resistance (R_CT_) in the Randles-Ershler equivalent circuit model. Z_W_ was extracted from the Nyquist plots using Equation (2). The phase change of the constant phase element α was calculated from the low-frequency slope. The peak frequency corresponding to the peak of the Nyquist semicircle value was used to determine C_G_, the geometric capacitance (Equation (2)). MATLAB was used to extract parameters based on the shape of the Nyquist plot. Real data is shown in Figure 3c for device 5 tested with a 100 pg/L αSyn constant ionic concentration solution.

Figure 3d shows unfiltered data for dilutions of αSynuclein in DI, showing a non-ideal Randles-Ershler impedance output curve for non-faradaic electrolytes under AC. The data trend shows a smaller semicircular curve and lower maximum real and imaginary capacitances for increasing concentration. There are two distinct behaviors that contribute to the shapes of the graph: increasing concentration and increasing binding. As R_S_ is a factor of solution concentration, it will decrease with increasing concentration of charged materials. With the increasing concentration, there is an increasing contribution to C_G_ from an increasing double-layer capacitance, with decreasing capacitance from an increase in binding to the surface. There are multiple explanations for the decrease in capacitance with increasing binding. It could be that the presence of proteins changes the conductivity in the near-surface region, the binding interrupts the formation of the EDL, or it could change the surface energy of the insulator [29]. The effect can be seen in the decreasing size of the semicircular portions of the Nyquist plots and the increasing impedance with increasing concentration. In order to observe the effects of binding alone, the ionic concentration of solutions was kept constant.

### 3.2. Characterizing Sensor Performance in

**Figure 4:**
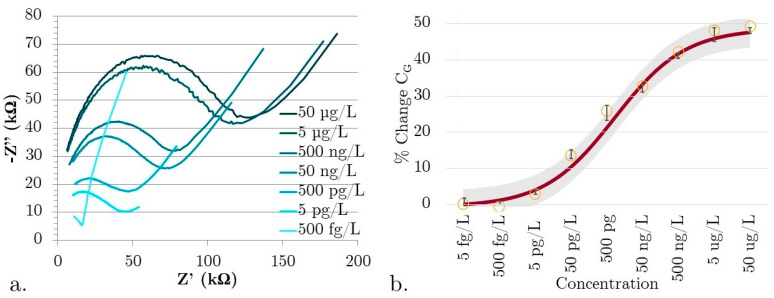
(**a**) Raw impedance data for the total synuclein protein concentration of 100 µg/L with decreasing ratios of αSyn to ßSyn (**b**) αSyn concentration dependence in constant ionic concentration solutions. Solid line is the fitted curve with grey region indicating the 95% confidence interval, circles are the averaged % Change C_G_ data with error bars showing the standard deviation.

Figure 4a shows real, unfiltered Nyquist plots showing the concentration-dependent change in C_G_ for our PCL SIP EIS biosensors tested in a constant ionic concentration environment with varying concentrations of αSyn. The solutions all have a total synuclein protein concentration of 100 µg/L but with a decreasing ratio of αSyn to ßSyn. The purpose of testing only in a constant ionic concentration environment is that these devices do have a non-specific response to electrolyte concentration. ßSyn is a homologous protein to αSyn that is structurally different, making it ideal as a control. As the ionic concentration remains constant, the capacitance change will be from increasing binding.

Plotting the Nyquist data clearly shows that not only is there a change in peak frequency (a dependent variable of C_G_), but the R_S_ is clearly increasing with concentration. This is a good indicator that there are indeed changes occurring at the surface of the biosensor. As these devices are tested in an aqueous environment under an applied bias, the electrolyte forms an EDL. The freely moving materials in the electrolyte align themselves to the surface. The effective thicknesses of these layers are on the Angstrom to nm level. As the non-Faradaic EIS biosensors do not have any charge transfer, the biosensing mechanism is due to changes in the EDL capacitance. With αSyn binding into the stereo cavities, the development of the EDL is interrupted, which decreases the capacitance. The impact of binding is great because the nm and Angstrom scale thicknesses of the EDL layers make the EIS SIP sensitive to near-surface interactions.

The concentration to percent change in geometric capacitance is shown in Figure 4b. The data was normalized across the five different sensor devices, with a 95% confidence interval. Parameters were extracted from the plots using the Randles-Erschler equivalent circuit model and fitted to a four-parameter logistical curve. Data is normalized using the 500 fg/L concentration test completed on each device prior to experimental data collection to allow for comparison between tested SIP EIS devices. LoD was determined by linear fitting using the standard method using LoD = 3.3(Sy/S), where Sy is the standard deviation of the sensor response extracted using linearly fitted data, and S is the slope of the sensor calibration curve [32]. In a simplistic estimation, concentrations that deviate by more than three standard deviations are considered outside of the linear detectable range. The biosensor has a linear range of 5 pg/L to 5 µg/L, with a LoD of 5 pg/L. The good resolution and concentration-dependent change in geometric capacitance demonstrate the potential for simple, soft-printed SIP biosensors as a breakthrough technology for low-concentration biomolecule detection. By testing the baseline geometric capacitance of our biosensor with TAE buffer throughout the testing process, we found a low standard variation of 7.2% (n = 12), indicating that the dilute acid and DI wash are effective in regenerating the PCL SIP surface by removing bound αSyn.

### 3.3. Preliminary Data from Microfluidic Channel SIP EIS Biosensor

**Figure 5:**
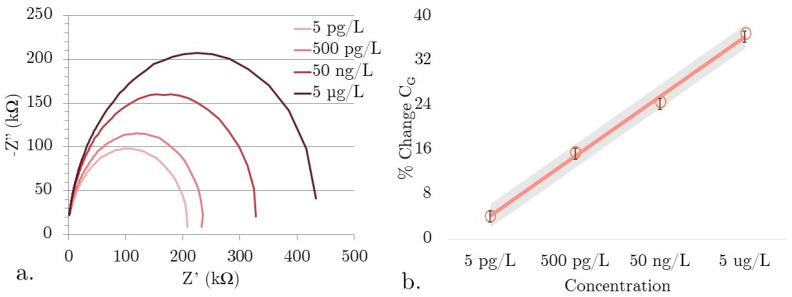
(**a**) Raw impedance data for series dilutions of αSyn in the total synuclein protein concentration in a 100 µg/L solution, (**b**) linear fit graph showing the demonstrable linear range response to αSyn for the microfluidic channel integrated SIP EIS biosensor. Solid line is the fitted linear curve with grey region indicating the 95% confidence interval, circles are the averaged % Change C_G_ data with error bars showing the standard deviation.

The final test was to create an EIS SIP biosensor device with the sensing area enclosed within a PCL microfluidic channel, with luer lock interconnects for efficient sample handling. The test volume of the PCL microfluidic channel device was kept at 100 µL. Repeating the same constant ionic concentration testing as with the previous device (shown in Figure 4) produced the data shown in Figure 5a,b.

The device was tested in the linear range established with the open-face biosensor. The EIS SIPs showed the same linear behavior and range enclosed in a microfluidic channel as when tested on an open surface, a highly desirable outcome (Figure 5b). It also highlights a source of work for the future, as enclosing the sensor in the microfluidic channel affects the change in geometric capacitance. The percent change in C_G_ decreased over the same linear range when the microfluidic channel was enclosed. Another significant change is the Nyquist plot shape. The absence of the low-frequency linear range can be explained by the enclosed channel minimizing dielectrophoretic droplet spreading. Investigating the bulk effects from the microfluidic channel will be the next stage of device development.

The PCL SIP has been demonstrated as a promising alternative to conventional affinity techniques. The effective and specific detection of αSyn in combined solutions demonstrates the comparability between SIP and conventional affinity techniques without the need for expensive extraction from animal models or complex aptamer development. The SIP EIS biosensor has a highly simple fabrication procedure that is desirable for rapid prototyping or large-scale manufacture. The LOD is lower than the dilute levels of αSyn in saliva, making this a potential application for these biosensors in the future. Saliva has a non-invasive extraction procedure, with fewer risk factors than serum or cerebral spinal fluid, and it can be repeatedly sampled [33]. Furthermore, the sensor can be easily interfaced with off-the-shelf portable EIS readers, making it point-of-care ready. Previously, we reported a MIP EIS sensor integrated with a PCB for portable and quantitative analysis of 8-Isoprostane in exhaled breadth [14]. Similarly, integrating the proposed SIP EIS is being adopted in the next phase of platform testing and validation.

The PCL SIP EIS biosensor has two main areas of work: optimizing the surface density and distribution of binding sites and investigating the behavior of the biosensor in biological samples. The distribution and density of binding sites impact the device-to-device variation and the signal-to-noise ratio. The testing in biological samples such as whole blood, serum, or interstitial fluid will investigate how the SIP performs in complex media where high-energy media components can interact with the simple binding sites that mainly use steric forces to select biomarkers of interest.

## 4. Conclusions

αSyn is a key biomarker for Parkinson’s Disease, a condition that presently lacks a non-invasive and accessible biomarker diagnostic method. To meet this, we have designed the PCL-based SIP EIS biosensor for αSyn, fabricated using a processable, low-temperature soft imprinting process. The benefits of our EIS biosensor are a scalable printing process, environmental stability of the PCL-based SIP bioreceptor surface, and a large linear detection range from 5 pg/L to 5 µg/L, which covers the physiological concentration range of αSyn in saliva samples. The sensor LoD was measured to be 5 pg/L, which is comparable to that of αSyn biosensors that rely on more expensive and less scalable receptors such as antibodies and aptamers. The regenerative capabilities of the PCL SIP surface make this device suitable for rapid and repeated testing of the biomarker. The biosensor testing using constant ionic concentration solutions of αSyn and ßSyn, a comparable synuclein protein, demonstrated no concentration-dependent behavior for ßSyn, confirming the specificity of this biosensor towards the target protein, i.e., monomeric αSyn protein. These outcomes make the PCL-based SIP EIS biosensor a highly promising method of quantifying pathogenic forms of αSyn monomers in clinical biofluid samples such as saliva and serum in future applications.

## Data Availability

The data presented in this study are available on request from the corresponding author.

## References

[1] Deuschl G., Beghi E., Fazekas F., Varga T., Christoforidi K.A., Sipido E., Bassetti C.L., Vos T., Feigin V.L. (2020). The burden of neurological diseases in Europe: An analysis for the Global Burden of Disease Study 2017. Lancet Public Health.

[2] (2017). Global Action Plan on the Public Health Response to Dementia. Geneva. https://iris.who.int/bitstream/handle/10665/259615/9789241513487-eng.pdf?sequence=1.

[3] Dorsey E.R., Glidden A.M., Holloway M.R., Birbeck G.L., Schwamm L.H. (2018). Teleneurology and mobile technologies: The future of neurological care. Nat. Rev. Neurol..

[4] Grimes D., Fitzpatrick M., Gordon J., Miyasaki J., Fon E.A., Schlossmacher M., Suchowersky O., Rajput A., Lafontaine A.L., Mestre T. (2019). Canadian guideline for Parkinson disease. Can. Med Assoc. J..

[5] Chung H.K., Ho H.-A., Pérez-Acuña D., Lee S.-J. (2019). Modeling α-Synuclein Propagation with Preformed Fibril Injections. J. Mov. Disord..

[6] Li A., Rastegar C., Mao X. (2022). α-Synuclein Conformational Plasticity: Physiologic States, Pathologic Strains, and Biotechnological Applications. Biomolecules.

[7] Meade R.M., Fairlie D.P., Mason J.M. (2019). Alpha-synuclein structure and Parkinson’s disease—Lessons and emerging principles. Mol. Neurodegener..

[8] Sorrentino Z.A., Giasson B.I. (2020). The emerging role of α-synuclein truncation in aggregation and disease. J. Biol. Chem..

[9] Russo M.J., Orru C.D., Concha-Marambio L., Giaisi S., Groveman B.R., Farris C.M., Holguin B., Hughson A.G., LaFontant D.-E., Caspell-Garcia C. (2021). High diagnostic performance of independent alpha-synuclein seed amplification assays for detection of early Parkinson’s disease. Acta Neuropathol. Commun..

[10] Orrù C.D., Ma T.C., Hughson A.G., Groveman B.R., Srivastava A., Galasko D., Angers R., Downey P., Crawford K., Hutten S.J. (2020). A rapid α-synuclein seed assay of Parkinson’s disease CSF panel shows high diagnostic accuracy. Ann. Clin. Transl. Neurol..

[11] Massey R.S., McConnell E.M., Chan D., Holahan M.R., DeRosa M.C., Prakash R. (2023). Non-invasive Monitoring of Alpha-Synuclein in Saliva for Parkinson’s Disease using Organic Electrolyte Gated FET Aptasensor. ACS Sensors.

[12] Adam H., Gopinath S.C., Arshad M.M., Parmin N., Hashim U. (2021). Distinguishing normal and aggregated alpha-synuclein interaction on gold nanorod incorporated zinc oxide nanocomposite by electrochemical technique. Int. J. Biol. Macromol..

[13] Smolinska-Kempisty K., Guerreiro A., Canfarotta F., Cáceres C., Whitcombe M.J., Piletsky S. (2016). A comparison of the performance of molecularly imprinted polymer nanoparticles for small molecule targets and antibodies in the ELISA format. Sci. Rep..

[14] Massey R.S., Gamero B., Prakash R. A System-on-Board Integrated Multi-analyte PoC Biosensor for Combined Analysis of Saliva and Exhaled Breath. Proceedings of the Annual International Conference of the IEEE Engineering in Medicine and Biology Society, EMBS.

[15] Di Mari G.M., Scuderi M., Lanza G., Salluzzo M.G., Salemi M., Caraci F., Bruno E., Strano V., Mirabella S., Scandurra A. (2024). Pain-Free Alpha-Synuclein Detection by Low-Cost Hierarchical Nanowire Based Electrode. Nanomaterials.

[16] Karaboğa M.N.S., Sezgintürk M.K. (2018). Cerebrospinal fluid levels of alpha-synuclein measured using a poly-glutamic acid-modified gold nanoparticle-doped disposable neuro-biosensor system. Analyst.

[17] Sande M.G., Rodrigues J.L., Ferreira D., Silva C.J., Rodrigues L.R. (2021). Novel Biorecognition Elements against Pathogens in the Design of State-of-the-Art Diagnostics. Biosensors.

[18] Unger C., Lieberzeit P.A. (2021). Molecularly imprinted thin film surfaces in sensing: Chances and challenges. React. Funct. Polym..

[19] Yang H., Song H., Suo Z., Li F., Jin Q., Zhu X., Chen Q. (2022). A molecularly imprinted electrochemical sensor based on surface imprinted polymerization and boric acid affinity for selective and sensitive detection of P-glycoproteins. Anal. Chim. Acta.

[20] Werner M., Glück M.S., Braeuer B., Bismarck A., Lieberzeit P.A. (2022). Investigations on sub-structures within cavities of surface imprinted polymers using AFM and PF-QNM. Soft Matter.

[21] El-Schich Z., Zhang Y., Feith M., Beyer S., Sternbæk L., Ohlsson L., Stollenwerk M., Wingren A.G. (2020). Molecularly imprinted polymers in biological applications. BioTechniques.

[22] Aguilar S.M., Shea J.D., Al-Joumayly M.A., Van Veen B.D., Behdad N., Hagness S.C. (2011). Dielectric Characterization of PCL-Based Thermoplastic Materials for Microwave Diagnostic and Therapeutic Applications. IEEE Trans. Biomed. Eng..

[23] Luo C.J., Stride E., Edirisinghe M. (2012). Mapping the influence of solubility and dielectric constant on electrospinning polycaprolactone solutions. Macromolecules.

[24] Malik N., Shrivastava S., Ghosh S.B. (2018). Moisture Absorption Behaviour of Biopolymer Polycapralactone (PCL)/Organo Modified Montmorillonite Clay (OMMT) biocomposite films. IOP Conf. Ser. Mater. Sci. Eng..

[25] Massey R.S., Li Y.H., Prakash R. Surface Imprinted Electroimpedance Biosensor for Detecting α-Synuclein for Parkinson’s Disease. Proceedings of the 2023 IEEE Sensors Applications Symposium (SAS).

[26] Barba L., Paoletti F.P., Bellomo G., Gaetani L., Halbgebauer S., Oeckl P., Otto M., Parnetti L. (2022). Alpha and Beta Synucleins: From Pathophysiology to Clinical Application as Biomarkers. Mov. Disord..

[27] Chae M.-S., Park J.H., Son H.W., Hwang K.S., Kim T.G. (2018). IGZO-based electrolyte-gated field-effect transistor for in situ biological sensing platform. Sensors Actuators B Chem..

[28] Magar H.S., Hassan R.Y.A., Mulchandani A. (2021). Electrochemical Impedance Spectroscopy (EIS): Principles, Construction, and Biosensing Applications. Sensors.

[29] Daniels J.S., Pourmand N. (2007). Label-Free Impedance Biosensors: Opportunities and Challenges. Electroanalysis.

[30] Tanak A.S., Jagannath B., Tamrakar Y., Muthukumar S., Prasad S. (2019). Non-faradaic electrochemical impedimetric profiling of procalcitonin and C-reactive protein as a dual marker biosensor for early sepsis detection. Anal. Chim. Acta X.

[31] Cruz-Manzo S., Greenwood P. (2020). An impedance model based on a transmission line circuit and a frequency dispersion Warburg component for the study of EIS in Li-ion batteries. J. Electroanal. Chem..

[32] Armbruster D.A., Pry T. (2008). Limit of blank, limit of detection and limit of quantitation. Clin. Biochem. Rev..

[33] Gröschl M. (2017). Saliva: A reliable sample matrix in bioanalytics. Bioanalysis.

